# Prevalence of abnormal uterine bleeding and quality of life after venous thromboembolism by oral anticoagulant use: the GENB-OAC Study

**DOI:** 10.1016/j.rpth.2025.103328

**Published:** 2025-12-31

**Authors:** Gabrielle Sarlon-Bartoli, Barbara Leclercq, Nathalie Trillot, Isabelle Mahé, Marie Daoud-Elias, Andrea Buchmuller, Geraldine Poenou, Antoine Elias, Jean Noel Poggi, Francis Couturaud, Noemie Resseguier, Martin Postzich, Lylia Hammoudi, Yasmine Benredouane, Florence Bretelle, Louisa Goumidi, Pierre Suchon, Sarah Jidal, Antonia Perez-Martin, Clementine Rousselin, Isabelle Quere, Sandrine Mestre, Marie Antoinette Sevestre, Simon Soudet, Laurent Bertoletti, Sophie Susen, Pierre Emmanuel Morange

**Affiliations:** 1 Aix Marseille Univ, INSERM 1263, INRAE 1260, C2VN and University Hospital of Marseille, Marseille, France; 2 Vascular Medicine and Hypertension Department, Assistance Publique Hôpitaux de Marseille - Hôpital de la Timone, Marseille, France; 3 FCRIN INNOVTE network, Saint Etienne, France; 4 Institut d'Hématologie-Transfusion, Centre Hospitalier Universitaire Régional de Lille, Lille, France; 5 Paris Cité University, Assistance Publique des Hôpitaux de Paris, Louis Mourier Hospital, Department of Internal Medicine, INSERM UMR_S1140, Innovations Thérapeutiques en Hémostase, Paris, France; 6 Department of Vascular Medicine, Centre Hospitalier Toulon La Seyne, Toulon, France; 7 Department of Medecine Vasculaire et Thérapeutique, Université Jean Monnet Saint Etienne, CHU Saint Etienne, Mines Saint Etienne, INSERM, SAINBIOSE U1059, CIC 1408, Saint Etienne, France; 8 Département de Médecine Interne et Pneumologie, CHU Brest, and Univ Brest, INSERM U1304-GETBO, CIC INSERM 1412, Brest, France; 9 Aix-Marseille University, Support Unit for Clinical Research and Economic Evaluation, Assistance Publique-Hôpitaux de Marseille, EA 3279 CEReSS-Health Service Research and Quality of Life Center, Marseille, France; 10 Service d’hématologie, Faculté de Médecine de Marseille, Aix-Marseille Université, Assistance Publique Hôpitaux de Marseille - Hôpital de la Timone, Marseille, France; 11 Department of Gynaecology and Obstetrics, Pôle Femme Enfant, AP-HM, Assistance Publique-Hôpitaux de Marseille, AMU, Aix-Marseille Université, Marseille, France; 12 IDESP, INSERM, Univ Montpellier, Service d’Exploration et de Médecin Vasculaire, CHU Nîmes, Nîmes, France; 13 Service de Médecine Interne et Vasculaire, Centre Hospitalier de Valenciennes, Valenciennes, France; 14 Department of Vascular Medicine, Centre de Référence des Maladies Lymphatiques et Vasculaires Rares, INSERM IDESP, CHU Montpellier, Université de Montpellier, Montpellier, France; 15 Department of Vascular Medicine, EA CHIMERE 7516, Picardie Jules Verne University, CHU Amiens Picardie, Amiens, France

**Keywords:** anticoagulants/adverse effects, female, quality of life, uterine hemorrhage, venous thromboembolism

## Abstract

**Background:**

Anticoagulants cause abnormal uterine bleeding (AUB) in women of reproductive age with venous thromboembolism, but the safety profiles of oral anticoagulants (OACs) in this setting are unclear.

**Objectives:**

To analyze and compare the prevalence of AUB and quality of life (QoL) in 4 groups (rivaroxaban, apixaban, vitamin K antagonists [VKAs], and controls).

**Methods:**

The GENital Bleeding Oral AntiCoagulant (GENB-OAC) study was a national, multicenter, observational, cross-sectional study conducted in 10 French hospitals from 2018 to 2022. The primary outcome was the proportion of women with major genital bleeding and/or clinically relevant non-major genital bleeding and/or pictorial blood loss assessment chart score >100.

**Results:**

Overall, 445 women were included: 122 on apixaban, 123 on rivaroxaban, 81 taking VKAs, and 119 healthy controls. The primary genital bleeding endpoint was significantly higher in OAC vs control group (94.8% vs 82.4%; *P* < .001) and the rivaroxaban or VKA vs apixaban group (96.7% or 97.5% vs 90.1%; *P* = .04 and *P* = .047). Major genital bleeding was similar in the apixaban and rivaroxaban groups, but menstruation ≥8 days, clinically relevant non-major bleeding, and pictorial blood loss assessment chart score >100 were significantly higher in the rivaroxaban vs apixaban group. QoL was significantly lower in the OAC than in the control group but was similar in the 3 OAC groups.

**Conclusion:**

AUB is frequent in women of reproductive age. OACs increase AUB and impact women’s QoL. Apixaban is associated with less AUB than rivaroxaban or VKAs with no difference in QoL. An international consensus is necessary to help clinicians detect and treat AUB in OAC users.

## Essentials

• Oral anticoagulants increase abnormal uterine bleeding in childbearing women.
• Oral anticoagulants worsen quality of life due to abnormal uterine bleeding.
• Apixaban causes less abnormal uterine bleeding than rivaroxaban or vitamin K antagonists.
• A simple definition is needed to detect and manage abnormal uterine bleeding.

## 1 Introduction

Abnormal uterine bleeding (AUB) is underestimated in the general population and is a potential complication of anticoagulant therapy in women of reproductive age affected with venous thromboembolism (VTE). However, only a few studies are available, and these have very heterogeneous definitions and prevalences of AUB. In randomized controlled therapeutic trials, only post-hoc studies are available. A post-hoc analysis of the EINSTEIN study was used to compare the incidence of recurrent VTE and AUB in women with and without concomitant hormonal therapy receiving rivaroxaban or enoxaparin/vitamin K antagonist (VKA) for VTE [1]. Among a total of 1888 women, AUB occurred significantly more frequently with rivaroxaban than with enoxaparin/VKA (hazard ratio, 2.13; 95% CI, 1.57-2.89) [1]. However, the definition of AUB was imprecise [1]. In a similar post-hoc analysis of the AMPLIFY trial [2], 1122 women were treated with apixaban and 1106 with enoxaparin/warfarin. There were no significant differences in major bleeding (<0.1% vs 0%, respectively) or clinically relevant non-major (CRNM) vaginal bleeding (2.5% vs 2.1%, respectively; odds ratio, 1.2; 95% CI, 0.7-2.0). In this analysis, major bleeding was defined as uterine bleeding leading to ≥1 unit of blood transfusion, and CRNM bleeding was defined as menstrual bleeding for >7 days or anemia (hemoglobin <11.9 g/dL) [2].

Data from retrospective studies are also available and provide avenues for research. De Crem et al. [3] studied AUB in a retrospective study that included 104 women. Compared with VKAs, rivaroxaban was associated with significantly prolonged menstrual bleeding and more medical interventions. However, the definition of AUB was semiquantitative, using the Fédération Internationale de Gynécologie et d'Obstétrique (FIGO; International Federation of Gynecology and Obstetrics) classification. In a retrospective study, Myers and Webster [4] directly compared the prevalence of AUB in 96 women on rivaroxaban and 43 on apixaban. The definition of heavy menstrual bleeding (HMB) was the same as that used by De Crem et al. [3]. The calculated likelihood of menorrhagia was higher in the rivaroxaban group than in the apixaban group (hazard ratio, 2.69; 95% CI, 0.99-7.27) [4].

Studies on quality of life (QoL) are also available and very interesting. In 2022, the recent TEAM-VTE study defined AUB using pictorial blood loss assessment charts (PBACs) or self-reported AUB and QoL using the Menstrual Bleeding Questionnaire (MBQ) in 98 women at the initiation of an oral anticoagulant (OAC) for VTE [5]. It showed that, at the beginning of anticoagulation therapy, QoL is altered in women with new-onset AUB compared with those without AUB or with pre-existing AUB (mean MBQ increase of 5.1 points; 95% CI, 2.2-7.9). Recently, the PERIOD study showed that women receiving anticoagulation also reported worse bleeding-related QoL scores compared with women in the control group [6].

AUB is thus a known complication of OAC in women. However, the relative safety profiles of the available OACs in this setting is, as yet, unknown as no study has ever prospectively compared the prevalence of AUB between the different OACs with sufficient statistical power. Our objective was, therefore, to prospectively evaluate and compare with a precise definition the prevalence of AUB in French women of reproductive age taking an OAC for VTE (rivaroxaban, apixaban, or a VKA) and in healthy controls. We also aimed to assess the impact on QoL in these 4 groups and in the general French population of women of the same age.

## 2 Methods

### 2.1 Study design

The GENital Bleeding Oral AntiCoagulant (GENB-OAC) study was a French, multicenter, cross-sectional, observational study conducted in 10 French hospitals from December 2018 to December 2022. The study protocol was approved by the French national ethics committee (Reference CPP: 53/18_3), and the study is registered at clinicaltrials.gov (NCT03772366).

### 2.2 Patients

We prospectively included women aged ≥18 years who were of reproductive age, had active menstrual cycles, and had a first or recurrent confirmed VTE treated with an OAC (rivaroxaban, apixaban, or a VKA) regardless of the date of VTE. Excluded were pregnant women, perimenopausal patients, and those who refused consent. A control group of healthy women of reproductive age without VTE and who were not taking an OAC was included as a reference for the quantification of AUB in women without treatment. We decided to include this control group because there were no data on the epidemiology of AUB in France. All included patients provided informed consent. The patients were recruited from follow-up consultations with a specialist (hematologist, vascular doctor, or cardiologist). Most of the patients were followed in hemostasis consultations, which makes the presence of an underlying hemorrhagic disorder unlikely. The controls were mainly recruited among medical or paramedical professionals working in thrombosis-specialized departments, which ensured good awareness of the study objectives and facilitated their inclusion.

### 2.3 Treatments

We recruited women who were already taking OACs (in some cases for a long period), and the choice of anticoagulant was at the discretion of each patient’s clinician. Three OACs were compared, namely apixaban, rivaroxaban, and VKAs. Each agent was dosed according to the guidelines for the prescription and marketing of the products in the indication of VTE disease.

### 2.4 Design

**Figure 1:**
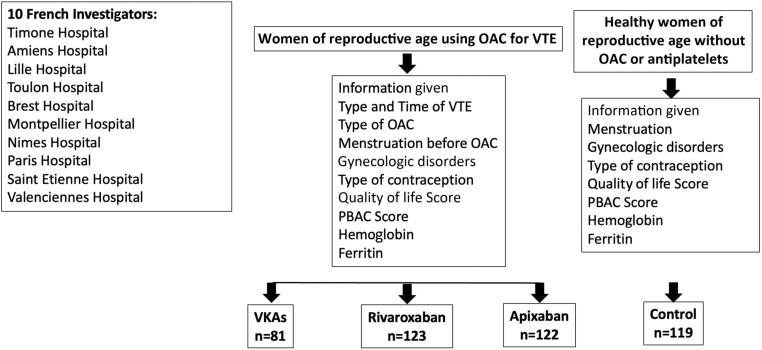
Study flowchart. OAC, oral anticoagulant; PBAC, pictorial blood assessment chart; VKA, vitamin K antagonist; VTE, venous thromboembolism.

The study flowchart is shown in Figure 1. Patients completed 2 PBAC scores with the medical doctor: 1 retrospectively for the period before they began taking an OAC and 1 during OAC use at the moment of the study inclusion, which did not always correspond to the time of anticoagulation initiation. The PBAC score is a method used to estimate menstrual blood loss in women experiencing heavy periods [7]. They also completed 1 World Health Organization Quality of Life Brief Version (WHOQOL-BREF) questionnaire during OAC use.

Biological samples were tested for hemoglobin, ferritin, and International Normalized Ratio (for those taking a VKA). Almost all women were tested for inherited thrombophilia, as French national recommendations advise thrombophilia screening in all women of childbearing age who have experienced a VTE event [8].

Data collected included type of VTE, bleeding history, type of contraception, gynecologic disease, and biological explorations. A gynecologist consultation was proposed for patients with major or CRNM genital hemorrhages.

### 2.5 Outcomes

The primary outcome combined different AUB assessment methods and was the proportion of women with major genital bleeding defined with criteria close to the International Society on Thrombosis and Haemostasis (ISTH) classification [9] (anemia [hemoglobin <12 g/dL], transfusion of ≥2 units) and/or CRNM genital bleeding defined with criteria close to the FIGO recommendations [10] and the ISTH classification [9] (menstrual periods lasting ≥8 days, intermenstrual bleeding, presence of blood clots, consultation with a healthcare professional, changing dose or stopping treatment) and/or minor bleeding using the PBAC semiquantitative score (>100 points, which is indicative of >80 mL of blood loss). Another analysis was conducted with a PBAC score >150 due to the high prevalence of PBAC score >100 in our population, as in the TEAM-VTE study [5]. Secondary outcomes were the components of the primary outcome. The term AUB was preferred to ‘menstrual bleeding’ because outcomes like anemia or transfusion or intermenstrual bleeding were larger than only menstrual bleeding.

QoL was measured using the WHOQOL-BREF scale [11], which includes 26 items with 5 responses. This gives 4 scores ranging from 0 to 100 (higher scores indicate better level of QoL) in the following 4 domains: physical health (how healthy and physically capable a person feels in their daily life), psychological health (how people think, feel, and view themselves and their lives), social relationships (how satisfied a person is with their social life and the support they receive from others), and environmental (how supportive and comfortable a person’s environment is for living a healthy and fulfilling life). QoL was compared between the OAC and control groups, between the 3 OAC groups, and to reference values of the WHOQOL-BREF scale in an age-matched female French population.

### 2.6 Statistical analysis

Baseline characteristics and endpoints related to bleeding and QoL were described and compared according to OAC vs control groups. Qualitative characteristics/endpoints were described as numbers and percentages and compared using chi-squared test if valid (Fisher’s exact test otherwise). Quantitative characteristics/endpoints were described as medians and IQRs and compared using Student’s *t*-test if valid (Mann–Whitney U-test otherwise). The QoL of each group was compared to age-matched reference values from the French female population using paired Student’s *t*-test.

A secondary exploratory analysis was performed by only considering the OAC group. Baseline characteristics and endpoints were described and compared according to the received molecule (apixaban, rivaroxaban, VKA). Data were first compared globally using chi-squared test if valid (Fisher’s exact test otherwise) for qualitative values and using analysis of variance if valid (Kruskal-Wallis test otherwise) for quantitative values. A post-hoc analysis was then performed by comparing the same data pairwise according to the groups defined by the received molecules. Chi-squared test was used for qualitative data if valid (Fisher’s exact test otherwise); Student’s *t*-test was used for quantitative data if valid (Kruskal-Wallis test otherwise).

Comparison of genital bleeding before and after anticoagulant therapy was performed in the overall OAC group and in each treatment group, using McNemar test for paired binary data. Considering conventional values for power at 80% and risk at 5%, a sample size of 124 women (per group) was determined to be necessary to detect intergroup difference at ±18%.

All statistical analyses were performed using R version 4.2.2. All tests were two-sided. Statistical significance was set at a threshold of *P* < .05.

## 3 Results

### 3.1 Comparison of OAC and control groups

A total of 445 women were included prospectively: 326 taking an OAC (122 apixaban, 123 rivaroxaban, and 81 VKAs) and 119 healthy controls. Among women taking an OAC, most had been diagnosed with a deep vein thrombosis (44.8%), followed by a pulmonary embolism (30.7%), or both (24.5%). This was a first episode of VTE for 55.8% of the patients, a risk factor was present for 63.8% (pregnancy 11.3%, surgery 9.8%, trauma 6.4%, cancer 1.2%, other minor factor 35.0%), and 38.0% of the women were taking the combined pill at the time of VTE diagnosis. Many (60.4%) had taken previous anticoagulant therapy before the treatment taken at inclusion, which they had stopped due to genital bleeding (4.3%, *n* = 14), other bleeding (0.9%), allergy (0.3%), or for other reasons such as a recurrent event, or simply for convenience (for example, the comfort of taking a single daily pill or avoiding biological monitoring) (54.9%).

Compared with the control group, women in the OAC group were older (mean age 36.4 vs 34.5 years; *P* = .04), had a higher mean body mass index (BMI) (28.0 vs 24.1 kg/m^2^; *P* < .001), were more likely to be smokers (21.2% vs 11.8%; *P* = .02), and were much more likely to have known hereditary thrombophilia (48.6% vs 0%; *P* < .001) (Table 1). Among those on contraceptives at inclusion, women taking an OAC were most likely to have a copper intrauterine device (IUD; 45.8%) or to be taking an oral progestogen (35.4%), while control women were most likely to be taking an oral estrogen (53.3%).

**Table 1:** Comparison of the OAC and control groups.

Characteristic	OAC (*n* = 326)	Control (*n* = 119)	*P*
Age, y, mean (SD)	36.4 (8.3)	34.5 (8.8)	.04
BMI, kg/m^2^, mean (SD)	28.0 (7.2)	24.1 (4.2)	<.001
Smoking, *n* (%)	69 (21.2)	14 (11.8)	.02
Hypertension, *n* (%)	20 (6.1)	2 (1.7)	.06
Diabetes, *n* (%)	9 (2.8)	3 (2.5)	1.00
Dyslipidemia, *n* (%)	7 (2.1)	4 (3.4)	.49
Stroke, *n* (%)	7 (2.1)	2 (1.7)	1.00
Coronary disease, *n* (%)	0 (0.0)	1 (0.8)	.27
Hereditary thrombophilia, *n*/*n* (%)	141/290 (48.6)	0/119 (0.0)	<.001
Antiphospholipid syndrome, *n*/*n* (%)	9/290 (3.1)	0/119 (0.0)	.06
Pregnancies, mean (SD)	1.7 (1.8)	1.4 (1.7)	.13
History of AUB^a^, *n* (%)	59 (18.1)	14 (11.8)	.11
Fibroma, *n* (%)	33 (10.1)	7 (5.9)	.17
Contraceptive type at inclusion, *n*/*n* (%)			<.001
Oral estrogen	13/96 (13.5)	24/45 (53.3)	
Oral progestogen	34/96 (35.4)	4/45 (8.9)	
Copper intrauterine device	44/96 (45.8)	13/45 (28.9)	
Hormone intrauterine device	3/96 (3.1)	4/45 (8.9)	
Other	2/96 (2.1)	0/45 (0.0)	
Hemoglobin, g/dL, mean (SD)	13.0 (1.4)	13.2 (1.1)	.32
Ferritin, ng/mL, median (IQR)	38.0 (58.1)	38.6 (59)	.37
Bleeding, *n* (%)			
Primary endpoint^b^ (PBAC >100)	309 (94.8)	98 (82.4)	<.001
As per the primary endpoint,^b^ but PBAC >150	301 (92.3)	92 (77.3)	<.001
Major genital bleeding^c^	53 (16.3)	8 (6.7)	.01
Anemia	53 (16.3)	8 (6.7)	.01
Transfusion	7 (2.1)	1 (0.8)	.69
CRNM genital bleeding^d^	277 (85.0)	76 (63.9)	<.001
Menstruation ≥8 d	60 (18.4)	11 (9.2)	.02
Blood clots	192 (58.9)	134 (41.1)	.002
Intermenstrual bleeding	58 (17.8)	12 (10)	.048
Presence of blood clots	192 (58.9)	51 (42.9)	.002
Consultation with a healthcare professional	74 (22.7)	23 (19.3)	.44
PBAC >100	279 (85.6)	74 (62.2)	<.001
PBAC >150	256 (78.5)	56 (47.1)	<.001

AUB, abnormal uterine bleeding; BMI, body mass index; CRNM, clinically relevant non-major; FIGO, Fédération Internationale de Gynécologie et d'Obstétrique (International Federation of Gynecology and Obstetrics); ISTH, International Society on Thrombosis and Haemostasis; OAC, oral anticoagulant; PBAC, pictorial blood assessment chart.

a History of AUB was defined to precedent AUB orally reported by the woman enrolled.

b Major genital bleeding according to the ISTH classification [9] (anemia [hemoglobin <12 g/dL], transfusion of ≥2 units) and/or CRNM genital bleeding according to the FIGO recommendations [10] (menstrual periods lasting ≥8 days, intermenstrual bleeding, presence of blood clots, consultation with a healthcare professional, changing dose, or stopping treatment) and/or minor bleeding using the PBAC semiquantitative score (>100 points).

c According to the ISTH classification [9] (anemia [hemoglobin <12 g/dL], transfusion of ≥2 units).

d According to the FIGO recommendations [10] (menstrual periods lasting ≥8 days, intermenstrual bleeding, presence of blood clots, consultation with a healthcare professional, changing dose, or stopping treatment).

The primary genital bleeding endpoint (major bleeding and/or CRNM bleeding and/or PBAC score >100) was significantly more common in the OAC than in the control group (94.8% vs 82.4%; *P <* .001), although we note that this was high in the control group (Table 1). Major genital bleeding occurred in 16.3% vs 6.7% (*P* = .01), CRNM genital bleeding in 85.0% vs 63.9% (*P* < .001), and PBAC score >100 in 85.6% vs 62.2% (*P* < .001).

### 3.2 Comparison between OAC groups

Of the 326 patients on an OAC, 122 were taking apixaban, 123 rivaroxaban, and 81 VKAs. Fourteen patients switched to another OAC due to genital bleeding before inclusion and were excluded from further analysis (11 apixaban, 1 rivaroxaban, and 2 VKA); hence, totals of 111, 122, and 79, respectively, were included in the 3 groups. Median durations of therapy (ie, the time between diagnosis of the last VTE and study inclusion) were 163 (IQR, 364.0) days for apixaban, 287.0 (IQR, 440.8) days for rivaroxaban, and 489 (IQR, 1261) days for VKAs (*P* < .01).

Women in the VKA group had the highest mean BMI (*P* = .005) and the highest proportions of inherited thrombophilia (*P* < .001) and antiphospholipid syndrome (*P* = .002) (Table 2).

**Table 2:** Comparisons between the anticoagulant therapy groups.

Characteristic	Apixaban (*n* = 111)	Rivaroxaban (*n* = 122)	VKAs (*n* = 79)	*P* (overall)	*P* (A vs R)	*P* (A vs VKA)	*P* (R vs VKA)
Age, y, mean (SD)	36.2 (8.4)	36.4 (9.0)	36.4 (7.4)	.98	.87	.84	.97
BMI, kg/m^2^, mean (SD)	26.4 (6.0)	28.5 (7.7)	29.8 (7.8)	.005	.02	.001	.26
Smoking, *n* (%)	19 (17.1)	28 (23.0)	18 (22.8)	.49	.27	.33	.98
Hypertension, *n* (%)	4 (3.6)	12 (9.8)	4 (5.1)	.13	.06	.62	.22
Diabetes, *n* (%)	5 (4.5)	1 (0.8)	3 (3.8)	.18	.10	1.00	.30
Dyslipidemia, *n* (%)	3 (2.7)	3 (2.5)	1 (1.3)	.90	1.00	.64	1.00
Stroke, *n* (%)	2 (1.8)	2 (1.6)	3 (3.8)	.63	1.00	.65	.38
Coronary disease, *n* (%)	0 (0.0)	0 (0.0)	0 (0.0)				
Hereditary thrombophilia, *n*/*n* (%)	30/92 (32.6)	54/111 (48.6)	52/75 (69.3)	<.001	.02	<.001	.005
Antiphospholipid syndrome, *n*/*n* (%)	0/92 (0.0)	2/111 (1.8)	7/75 (9.3)	.002	.50	.003	.31
Pregnancies, mean (SD)	1.6 (1.6)	1.8 (2.2)	1.6 (1.6)	.61	.37	.88	.50
History of AUB, *n* (%)	25 (22.5)	22 (18.0)	20 (25.3)	.44	.39	.65	.21
Fibroma, *n* (%)	11 (9.9)	13 (10.7)	6 (7.6)	.77	.85	.58	.47
Contraceptive type at inclusion, *n*/*n* (%)				.19	.78	.06	.22
Oral estrogen	5/32 (15.6)	7/34 (20.6)	1/25 (4.0)				
Oral progestogen	9/32 (28.1)	10/34 (29.4)	12/25 (48.0)				
Copper intrauterine device	18/32 (56.3)	15/34 (44.1)	9/25 (36.0)				
Hormone intrauterine device	0/32 (0.0)	1/34 (2.9)	2/25 (8.0)				
Other	0/32 (0.0)	1/34 (2.9)	1/25 (4.0)				
Hemoglobin, g/dL, mean (SD)	12.9 (1.4)	13.1 (1.3)	13.1 (1.4)	.50	.31	.31	.87
Ferritin, ng/mL, median (IQR)	36.90 (61.35)	34 (51.5)	49 (72)	.28	.75	.12	.25
Bleeding, *n* (%)							
Primary endpoint^a^ (PBAC >100)	100 (90.1)	118 (96.7)	77 (97.5)	.049	.04	.047	1.00
As per the primary endpoint,^a^ but PBAC >150	96 (86.5)	116 (95.1)	75 (94.9)	.03	.02	.06	1.00
Major genital bleeding^b^	17 (15.3)	17 (13.9)	14 (17.7)	.77	.77	.66	.47
Anemia	17 (15.3)	17 (13.9)	14 (17.7)	.77	.77	.66	.47
Transfusion	2 (1.8)	0 (0.0)	3 (3.8)	.07	.23	.65	.06
CRNM genital bleeding^c^	81 (73.0)	110 (90.2)	72 (91.1)	<.001	<.001	<.001	.81
Menstruation ≥8 days	10 (9.0)	26 (21.3)	21 (26.6)	.005	.01	.001	.39
Blood clots	63 (51.6)	82 (66.7)	47 (58.0)	.06	.02	.45	.27
PBAC >100	90 (81.1)	111 (91.0)	66 (83.5)	.08	.03	.66	.11
PBAC >150	81 (73.0)	102 (83.6)	61 (77.2)	.14	.048	.51	.26

A, apixaban; AUB, abnormal uterine bleeding; BMI, body mass index; CRNM, clinically relevant non-major; FIGO, Fédération Internationale de Gynécologie et d'Obstétrique (International Federation of Gynecology and Obstetrics); ISTH, International Society on Thrombosis and Haemostasis; PBAC, pictorial blood assessment chart; R, rivaroxaban; VKA, vitamin K antagonist.

a Major genital bleeding according to the ISTH classification [9] (anemia [hemoglobin <12 g/dL], transfusion of ≥2 units) and/or CRNM genital bleeding according to the FIGO recommendations [10] (menstrual periods lasting ≥8 days, intermenstrual bleeding, presence of blood clots, consultation with a healthcare professional, changing dose, or stopping treatment) and/or minor bleeding using the PBAC semi-quantitative score (>150 or >100 points).

b According to the ISTH classification [9] (anemia [hemoglobin <12 g/dL], transfusion of ≥2 units).

c According to the FIGO recommendations [10] (menstrual periods lasting ≥8 days, intermenstrual bleeding, presence of blood clots, consultation with a healthcare professional, changing dose, or stopping treatment).

**Figure 2:**
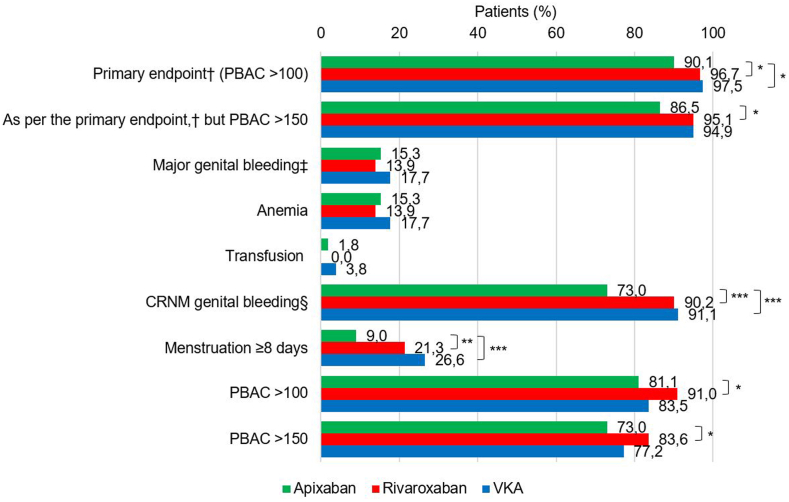
Bleeding outcomes in the 3 anticoagulant therapy groups. ∗*P* < .05; ∗∗*P* ≤ .01; ∗∗∗*P* ≤ .001. ^†^Major genital bleeding according to the ISTH classification [9] (anemia [hemoglobin <12 g/dL], transfusion of ≥2 units) and/or CRNM genital bleeding according to the FIGO recommendations [10] (menstrual periods lasting ≥8 days, intermenstrual bleeding, presence of blood clots, consultation with a healthcare professional, changing dose, or stopping treatment) and/or minor bleeding using the PBAC semiquantitative score (>150 or >100 points). ^‡^According to the ISTH classification [9] (anemia [hemoglobin <12 g/dL], transfusion of ≥2 units). ^§^According to the FIGO recommendations [10] (menstrual periods lasting ≥8 days, intermenstrual bleeding, presence of blood clots, consultation with a healthcare professional, changing dose, or stopping treatment). CRNM, clinically relevant non-major; FIGO, Fédération Internationale de Gynécologie et d'Obstétrique (International Federation of Gynecology and Obstetrics); ISTH, International Society on Thrombosis and Haemostasis; PBAC, pictorial blood assessment chart; VKA, vitamin K antagonist.

The primary genital bleeding endpoint was significantly lower with apixaban vs rivaroxaban or VKAs (90.1% vs 96.7% and 97.5%, respectively; *P* = .04 and *P* = .047; Figure 2 and Table 2). Patients taking apixaban were also less likely to have CRNM genital bleeding (73.0% vs 90.2% and 91.1%, respectively; both *P* < .001) and prolonged menstruation (9.0% vs 21.3% and 26.6%; *P* = .01 and *P* = .001). There was no statistically significant difference for major genital bleeding according to the timing of the VTE event, regardless of the anticoagulant used (*P* = .52).

### 3.3 Comparison of genital bleeding before and after anticoagulant therapy

**Figure 3:**
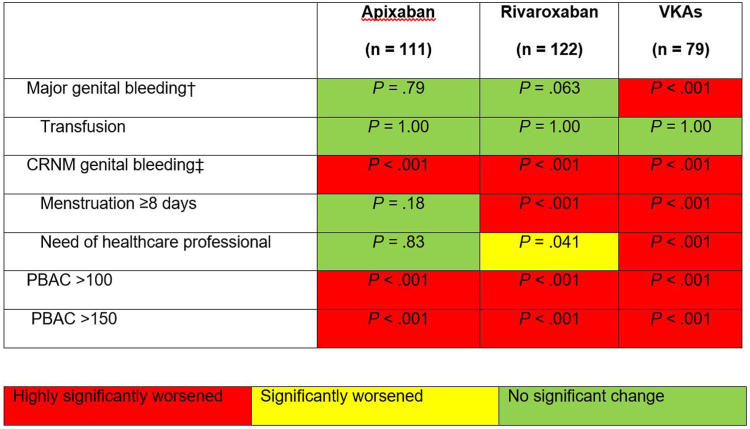
Comparison of AUB before vs after anticoagulant therapy. ^†^According to the ISTH classification [9] (anemia [hemoglobin <12 g/dL], transfusion of ≥2 units). ^‡^According to the FIGO recommendations [10] (menstrual periods lasting ≥8 days, intermenstrual bleeding, presence of blood clots, consultation with a healthcare professional, changing dose, or stopping treatment). AUB, abnormal uterine bleeding; CRNM, clinically relevant non-major; FIGO, Fédération Internationale de Gynécologie et d'Obstétrique (International Federation of Gynecology and Obstetrics); ISTH, International Society on Thrombosis and Haemostasis; PBAC, pictorial blood assessment chart; VKA, vitamin K antagonist.

The retrospectively estimated mean ± SD PBAC score before OAC was 142.6 ± 174 in the apixaban group), 147.5 ± 165.3 in the rivaroxaban group, and 182.2 ± 160 in the VKA group (*P* = .24). We compared genital bleeding before and after initiation of anticoagulant therapy for the 7 available variables: major genital bleeding, transfusion, CRNM genital bleeding, menstruation ≥8 days, need for a healthcare professional, PBAC score >100, and PBAC score >150. In fact, we did not have the quantitative variable on hemoglobin before anticoagulant therapy. In the apixaban group, there was no significant change for 4 of the 7 genital bleeding variables available, while 3 worsened significantly (Figure 3). In the rivaroxaban group, only 2 variables had no significant change, and 5 worsened significantly, whereas in the VKA group, 6 of 7 variables worsened significantly.

### 3.4 Quality of life

**Figure 4:**
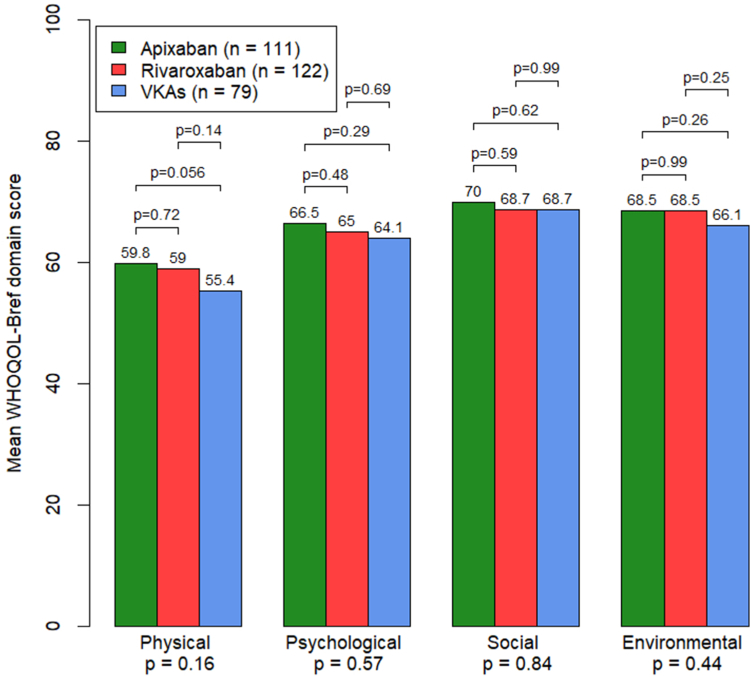
Comparison of WHOQOL-BREF domains between the OAC and control groups and French women. OAC, oral anticoagulant; VKA, vitamin K antagonist; WHOQOL-BREF, World Health Organization Quality of Life Brief Version.

The 4 WHOQOL-BREF domain scores were significantly lower in the OAC than the control group (Figure 4). In the OAC group, the physical and social domain scores were significantly lower than reference scores for an age-matched French female population. In the control group, the physical and social domain scores were similar to those of the French female population, while the psychological domain score was better.

**Figure 5:**
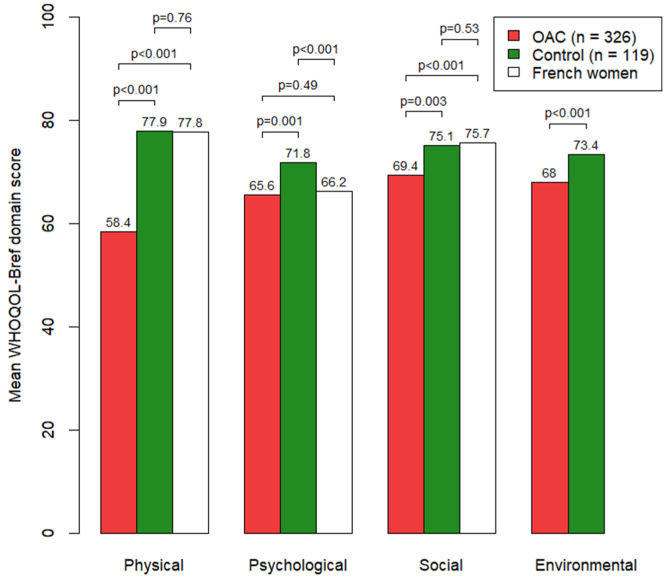
Comparison of WHOQOL-BREF domains by OAC group. OAC, oral anticoagulant; VKA, vitamin K antagonist; WHOQOL-BREF, World Health Organization Quality of Life Brief Version.

There were no significant differences in WHOQOL-BREF domain scores between the 3 OAC groups (Figure 5), although there was a trend toward better physical domain scores with apixaban vs VKAs (*P* = .06).

### 3.5 Variability of PBAC score depending on the inclusion center

**Figure 6:**
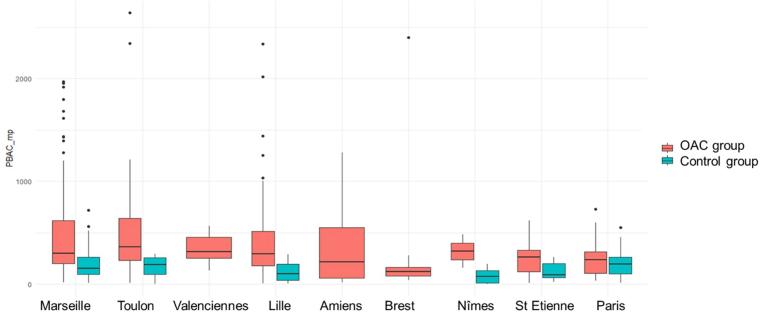
Distribution of PBAC score depending on center and group. OAC, oral anticoagulant; PBAC, pictorial blood assessment chart.

The distribution of PBAC score depending on inclusion center and group is presented in Figure 6. The mean PBAC score in control group was: 202.6 ± 151.1 in Marseille center, 167.8 ± 131.6 in Toulon center, 122.4 ± 116.8 in Lille center, 76.6 ± 67 in Nîmes center, 129.4 ± 80.3 in Saint Etienne center, and 208.3 ± 145.6 in Paris center. Valenciennes, Amiens, and Brest did not include patients in control group.

## 4 Discussion

This study is the first, to our knowledge, that was designed to directly compare AUB in women using different OACs. We found that apixaban used as an OAC for women with a VTE resulted in significantly less AUB than rivaroxaban or VKA, with no difference in QoL. There are no large direct randomized controlled trials comparing AUB in patients taking rivaroxaban or apixaban. The MEDEA study remains the only available randomized trial addressing this question; it was an open-label, pragmatic clinical trial to evaluate management strategies in premenopausal women with HMB associated with factor Xa inhibitor therapy [12]. Women using factor Xa inhibitors with proven HMB as evidenced by a single PBAC score ≥150 were randomized to 1 of 3 study arms: (1) switch to dabigatran; (2) continue factor Xa inhibitor with the addition of tranexamic acid during the menstrual period; or (3) continue factor Xa inhibitor without intervention. Unfortunately, due to a low recruitment rate, the study was terminated early. In both intervention groups, similar numeric trends toward reduced bleeding scores were observed, while the mean bleeding scores remained unchanged in the control group [13]. Data from small cohorts or retrospective studies have shown more AUB with rivaroxaban than with warfarin or apixaban [[1], [2], [3], [4]]. Our study confirms these results and shows that major genital bleeding is similar between the 3 different OACs but that menstruation ≥8 days, CRNM genital bleeding, and PBAC score >100 are significantly higher with rivaroxaban vs apixaban. Patients taking apixaban reported no worsening of lengthy menstruation or the use of a healthcare professional for treating AUB from before to after starting anticoagulant therapy, while these worsened for women on rivaroxaban or a VKA. A 2021 retrospective study of women taking apixaban, rivaroxaban, or warfarin found that women taking rivaroxaban were more likely to have AUB than those taking apixaban or warfarin [14]. Patients taking rivaroxaban also experienced longer menses and were more likely to require medical or surgical intervention [3]. Women who experienced AUB while taking rivaroxaban had up to a 5-fold increased risk of recurrent VTE compared with those who did not experience AUB, possibly due to increased rates of anticoagulation modification, including shortened courses and missed doses [3]. Moreover, our study included a relatively large number of patients on VKAs, and we did not observe any relevant differences in the AUB profile between VKA-treated women and those receiving direct OACs. The potential mechanistic differences (if any) between VKA and direct OACs in AUB therefore remain to be clarified.

In our cross-sectional, multicenter study of 445 adult women of reproductive age, AUB (defined as major bleeding and/or CRNM bleeding and/or PBAC >100) was frequent in the control group (82.4%) but was even more frequent among patients taking an OAC (94.8%). CRNM genital bleeding occurred in 63.9% of the control group, and a PBAC score >100 was observed in 62.2% of these participants. This extremely high rate is primarily due to the combination of multiple bleeding criteria, which increased the estimated prevalence. Some of these criteria were also subjective—such as menstrual duration or the presence of clots—which likely contributed to the elevated rate. For the most part, participants in our control group also work in the medical field of hemostasis and may have overestimated the prevalence of AUB due to high awareness of this problem. The symptom of AUB is one of the most common gynecologic complaints, and although data from healthcare systems suggest a prevalence of 3% to 5%, population-based studies using definitions like prolonged menstrual bleeding or alteration of QoL suggest that it may affect up to 50% of women of reproductive age [15]. AUB accounts for 18% to 30% of all gynecologic visits and leads to more than half of the 600,000 hysterectomies performed annually for benign disorders in the United States [16]. In a case-control study to assess the gynecologic experience of women with von Willebrand’s disease, 61% of the group control (*n* = 88) most commonly reported HMB [17]. These results highlight the challenges in defining AUB in women and the need to collectively develop a standardized method of assessment. Our findings support that using complex scores such as the PBAC may not be ideal, as they lack reproducibility, as suggested by Reid et al. [7]*,* and may lead to overestimation. In contrast, focusing on simpler variables, such as menstrual duration, could serve as a more practical and reliable approach.

The high prevalence of AUB in our control group was probably due to combination of our composite criteria, which increased the prevalence, and also, the high proportion of users of copper IUDs (28.9%) in the control group. To reiterate, our control group mostly works in the medical field of hemostasis, and women are probably more worried about taking hormones given the illnesses they treat because of estroprogestative drugs. In the latest epidemiologic data on contraception in France, 25.6% of the women used an IUD, with approximatively half of these using a copper device [18]. The use of the copper IUD is relatively common in France, partly due to a legal complaint filed by a young woman in 2012, which triggered a crisis of confidence among French women [19]. This mistrust is also observed in other Western countries, often driven by an excessive fear of hormones, rooted in the overestimation of associated health risks—frequently fueled by recurring media controversies surrounding hormonal contraception [19]. In any case, bleeding irregularities, such as intermenstrual spotting or heavy or prolonged menstrual bleeding, are common among copper-containing IUD users and are one of the leading reasons for method discontinuation [20]. All these data imply that these women's periods can be aberrant and affect their QoL, and a systematic assessment during a general medical and/or gynecologic consultation should be recommended to detect this reality and correct any iron deficiency.

The 4 QoL domains were worse in the patients taking an OAC compared with the controls. de Jong et al. [5] also found decreased QoL in women with AUB after OAC therapy using a specific QoL questionnaire (MBQ), but this decrease was observed among women with new-onset AUB after initiation of OAC. Recently, the PERIOD study showed that women taking anticoagulants reported significantly higher PBAC scores than controls (*P* < .05), with 60% to 64% vs 28% to 34% reporting HMB. Women in the anticoagulation group also reported worse bleeding-related QoL scores than women in the control group (*P* = .001) [6]. When comparing QoL scores across the OAC groups, we did not observe any statistically significant differences, likely due to the use of a generic questionnaire rather than a bleeding-specific tool such as the MBQ. However, patients taking apixaban showed numerically higher scores in the physical domain compared with those on VKAs (*P* = .56).

Our results are crucial to make clinicians aware of the problem of AUB among women taking OACs and to propose a consensus on how to detect and treat these complications. In 2017, Boonyawat et al. [21] proposed an algorithm for the treatment of AUB. Women who experience AUB should be referred to a gynecologist to screen for another cause of AUB, such as fibroma or cancer. Treatment options include the following: hormonal contraception (oral progesterone therapy, progesterone-containing IUD, or combined hormonal therapy); a change of OAC (modification of the molecule, dose reduction on the first day of menstruation, long-term dose reduction or stopping OAC because treatment is sufficient); treatment of iron deficiency; or giving an alternative therapy (tranexamic acid, surgery). Recently, we developed a prognostic score combining age, weight, uterine cancer, uterine bleeding history, anemia, estrogen-related VTE, and direct OAC use to help clinicians detect and treat AUB in women taking OACs [22]. In a recent review, Samuelson Bannow [23] summarized the incidence and complications of AUB in anticoagulated patients as well as management strategies for AUB in this population. She also addressed the patient experience, including the impact of HMB on QoL and the impact of discontinuing hormonal therapies at the time of VTE diagnosis and anticoagulant initiation [23]. Finally, Samuelson Bannow et al. [24] emphasized the importance of interdisciplinary management of gynecologic bleeding with anticoagulation with the role of various team members, including hematologists, gynecologists, adolescent medicine providers, and nurses, in providing comprehensive, interdisciplinary care to menstruating individuals. Our data suggest that apixaban should be the first-line OAC for women of reproductive age to reduce the potential bleeding risk. However, more prospective studies on this strategy are needed.

Our study has some limitations. First, patients were not included at the time of VTE; hence, our results could be heterogeneous because of missing potential data of the VTE and treatment history. Given the long duration of OAC use in most participants, recall bias or incomplete recollection cannot be ruled out (for example, forgetting certain details if thromboembolic event occurred a long time ago). In particular, the PBAC score prior to OAC initiation was calculated retrospectively, and the results may therefore be approximate and subject to recall bias. However, as this limitation likely impacted all OAC groups similarly, it should not have introduced a differential bias in the comparative analysis except for the VKA group. Second, this is a cross-sectional study; participants completed a single study visit during which they retrospectively reported their menstrual bleeding before and after initiating OAC. Patients were not followed, and this decision may also have led to the loss of potentially valuable data. Future studies should include longitudinal follow-up to better assess changes in bleeding patterns and QoL during anticoagulant treatment. Third, the control group was not entirely representative of the active group. People working in health care are possibly more educated and may have better life habits. Indeed, baseline characteristics show that patients in the control group smoke less and have a lower BMI. However, we chose these controls for feasibility reasons, mainly due to the ease of recruitment as they worked within our institution. One other limitation of this study is the absence of data on race/ethnicity, which could provide further insights into the sociocultural determinants of health. However, in accordance with the guidelines set by the French Data Protection Authority, sensitive data such as ethnic origin or immigration status were not collected. Given the lack of significant associations between ethnicity and key outcomes (eg, choice of anticoagulant, AUB events), we do not believe that the absence of this information substantially impacts the study’s conclusions. Also, we chose to use a precise definition of AUB based on multiple criteria, which may have been overly complex. CRNM genital bleeding yielded similar results to the PBAC score, although the latter can be difficult and time-consuming to implement in practice, with heterogenous results, as shown in Figure 6. Our findings suggest that using a simple criterion, such as prolonged menstruation lasting ≥8 days—as defined in our study—could be sufficient to assess AUB risk in clinical consultations (Figure 2). Also, we decided to use a nonspecific scale measuring QoL to appreciate the overall impact of AUB in women and to compare results to data available for the French population, but the use of a specific scale (eg, MBQ) might have enabled us to observe a difference in QoL between patients taking apixaban, rivaroxaban, and VKAs. We also decided to exclude 14 patients from the comparison of the 3 OAC groups because they changed OAC due to genital bleeding before inclusion, and this decision could have modified the results. Finally, potential confounding factors such as age, BMI, smoking status, and type of contraception were not included in the statistical models due to the cross-sectional design and sample size constraints. Future prospective studies with larger cohorts should include multivariable analyses to better account for these potential confounders.

In conclusion, the GENB-OAC study confirms that AUB is frequent in women of reproductive age, particularly those taking an OAC. Apixaban was associated with significantly less AUB than rivaroxaban, but there was no significant difference in nonspecific QoL scores. An international consensus is necessary to easily define AUB and to help clinicians to detect and treat this specific bleeding. A simple variable such as duration of period ≥8 days and/or alteration of specific QoL component could be a good tracking tool to detect AUB rather than complex scores like PBAC. Finally, it may also be beneficial to propose apixaban as first-line therapy for women of reproductive age.
